# Supplementary material for: Identification of photoperiod-induced specific miRNAs in the adrenal glands of Sunite sheep (Ovis aries)
Source: Front Vet Sci. 2022 Jul 22;9:888207. doi: 10.3389/fvets.2022.888207 (PMC9354845; doi:10.3389/fvets.2022.888207)
Supplement: Supplementary file 1 [file Data_Sheet_1.ZIP › Supplementary file/miRDeep2 file.zip!/miRDeep2 file/Know_Structure/LP42A3/oar-mir-150.pdf]

miRBase precursor : oar-mir-150
Total read count : 602
oar-miR-150 read count : 602
remaining reads : 0

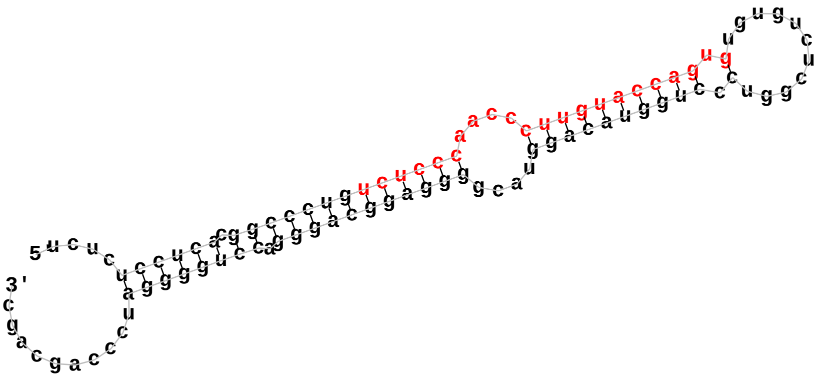

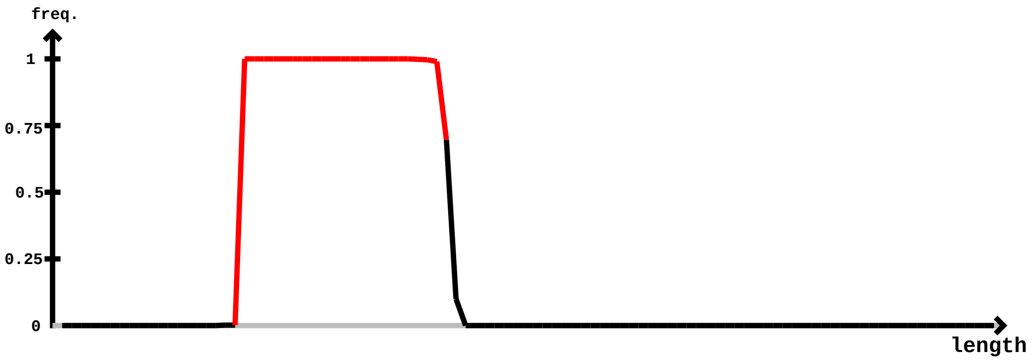


```
                  oar-miR-150
5'- ucucuccucacggcccugucucccaacccuuguaccagugugugucucggucccugguacagguacggggaggcagggaccuggggaucccagcagc   -3'    exp
    ....((((((.(((((((((((((....((((((((((.(...........).))))))))))....))))))))))).))))))))..........    reads      mm          sample
    ................ugucucccaacccuuguaccagu..........................................................    1          0           seq
    ..................ucucccaacccuuguacc.............................................................    1          0           seq
    ..................ucucccaacccuuguacca............................................................    1          0           seq
    ..................ucucccaacccuuguaccag...........................................................    4          0           seq
    ..................ucucccaacccuuguaccagu..........................................................    176        0           seq
    ..................ucucccaacccuuguaccagug.........................................................    359        0           seq
    ..................ucucccaacccuuguaccagugu........................................................    60         0           seq
```
